# Binary Binder for Cf/C-SiC Composites with Enhanced Mechanical Property

**DOI:** 10.3390/ma15082757

**Published:** 2022-04-08

**Authors:** Yun Liu, Long Ma, Runa Dong, Kexin Cui, Yongzhao Hou, Wen Yang, Yeqing Liu, Cheng Zhong, Guangwu Wen, Lijuan Zhang

**Affiliations:** 1 School of Materials Science and Engineering, Shandong University of Technology, Zibo 255000, China; ly17864301063@163.com (Y.L.); ml18340071781@163.com (L.M.); drn19121102039@163.com (R.D.); ckx9221@163.com (K.C.); aa1435884413@163.com (C.Z.); zhanglj@sdut.edu.cn (L.Z.); 2 Shandong Guiyuan Advanced Ceramics Co., Ltd., Zibo 255086, China; 3 School of Transportation and Vehicle Engineering, Shandong University of Technology, Zibo 255000, China; yangwen004@sdut.edu.cn; 4 Shandong Si-Nano Materials Technology Co., Ltd., Zibo 255400, China; liuyeqingyao@outlook.com

**Keywords:** Cf/C-SiC composite, binary binder, SiC precursor, coal pitch

## Abstract

Cf/C-SiC composites have become the preferred material for high-temperature load-bearing applications because of their low density, high strength, and excellent thermal-physical properties. Due to the composite’s poor sintering performance, the sintering temperature and pressure required for the preparation of Cf/C-SiC by traditional methods are also relatively high, which limits its engineering application. Herein, based on the precursor-derived ceramic route and C/C composites material preparation process, a binary binder (coal pitch and polysilylacetylene) is developed, which combines a carbon source, SiC precursor, and semi-ceramic SiC filler organically. Then, the SiC phase was successfully introduced into C/C composites by the slurry impregnation-hot pressing sintering method. The prepared Cf/C-SiC composites showed good mechanical properties, with a density of 1.53 g/cm^3^ and a bending strength of 339 ± 21 MPa. Moreover, the effects of the binary binder on the microstructure, density, and mechanical properties of Cf/C-SiC composites were investigated. This work provides a novel and effective approach to fabricating Cf/C-SiC composites with low density and high strength.

## 1. Introduction

Because of its low density and high specific strength [1,2,3], the carbon-fiber-reinforced carbon matrix composite (C/C composite) is widely used in aerospace and nuclear fields, such as the thermal structure for solid rocket engine exhaust, disc brake for aircraft and shell for nuclear reactor [4]. However, the oxidation of Cf/C composites and poor abrasion resistance of C/C composite affects the mechanical properties and service life, which greatly limits their application in extreme environment [5].

To overcome the above problems, ceramic materials have been combined with Cf/C composites [6], such as ZrC, ZrB_2_, SiC, TaC, and HfC. In particular, Silicon carbide (SiC) is considered a suitable reinforcing material for Cf/C composites because of its low thermal expansion coefficient (CTE, α-SiC = 4.6 × 10^−6^ K^−1^), good high-temperature stability, high hardness, excellent corrosion resistance, and thermal shock resistance [7,8]. However, the introduction of SiC and the combination between SiC and carbon matrix are the key problems for Cf/C-SiC composites.

In recent years, many methods were developed to introduce the SiC ceramic into the carbon matrix, for instance, (i) polymer infiltration and pyrolysis (PIP) [9,10], (ii) chemical vapor infiltration (CVI) [11], (iii) hot pressing (HP) [12], (iv) reactive melt infiltration (RMI) [13,14], (v) slurry infiltration, etc., or a combination of methods (e.g., CVI + PIP) [9,15]. The PIP method includes the synthesis, infiltration, and pyrolysis of SiC precursors, which need more than six cycles for the relatively dense composites [16]. The CVI method is considered a low-efficiency and high-cost process depending on the permeation and decomposition of gaseous molecules [17]. The RMI technology mainly involves the infiltration of molten or gaseous Si into the porous fiber-reinforced preform, which is difficult to control the residual silicon.

Compared with the above three methods, the HP sintering method is a low-cost and commercial-application technique, which uses a mixture of carbon fibers, SiC powders, and sintering additives. However, the sintering aids may remain at the grain boundaries, which has a negative impact on the properties of the composites. Recently, the precursor-derived ceramic route (PDC) is also available for introducing SiC ceramics into Cf/C composites during the HP method [18]. Compared with introducing SiC powder directly into Cf/C composites, this method greatly reduces the sintering temperature [19] and improves the distribution of SiC phases in the composites, especially in fiber bundles. Stalin et al. [20] prepared Cf/SiC composites by HP using precursor (polycarbosilane and polysilazane) as polymer binders. The flexural strength of 138 MPa was obtained in the case of Cf/SiC composite synthesized with 30 wt% initial PCS (polycarbosilane) content. Similarly, a carbon-based binder was widely used for Cf/C composites, which combined graphite, petroleum coke powder, and other aggregates during sintering [21]. Therefore, a novel binder (containing SiC precursors) could be available for Cf/C-SiC composites, which bonds aggregates and carbon fibers during the slurry impregnation process and converts to SiC during hot pressing sintering.

In this paper, the coal pitch and SiC precursor were mixed to prepare the binary binder for the first time, which not only improved the problem of rapid and concentrated air release interval of the original coal pitch binder but also introduced the SiC ceramic component. Cf/C-SiC composites with excellent mechanical properties were prepared at a low sintering temperature (1400 °C). The effect of the binary binder on the microstructure and mechanical properties of Cf/C-SiC composites was also discussed.

## 2. Materials and Methods

### 2.1. Materials

Petroleum coke was purchased from Beijing Boyu Gaoke New Materials Co., Ltd. (Beijing, China). Coal pitch was provided by Jining Carbon Group Co., Ltd. (Jining, China). Chemical reagents for PSA (polysilyacelene) synthesis were purchased from Aladdin, Shanghai, China and all other chemicals were purchased from the Inno-chem Co, Beijing, China. The carbon fiber was T300 3K, provided by Toray, Tokyo, Japan.

### 2.2. Synthesis of Binary Binder

The binary binder was prepared by mixing coal pitch and the PSA powder (produced in our lab, with a mean molecular weight of 1400) under a constant temperature. The overview of the forming process is shown in Figure S1. PSA resin was heated to 260 °C in an oil bath to obtain a liquid resin with good fluidity. With grinding and screening (100 mesh), coal pitch powder was gradually added to PSA. After fully stirring for 30 min and being cooled to room temperature, the binary binder was manufactured. This process eliminated a small part of small molecule volatiles, which increased the porosity during the carbonization and consequently affected the mechanical properties of the composites. Additionally, the thermal treatment helped to improve the consistency of the binary binder, which was crucial for the hot compression process in the presence of a chemically treated binary binder. According to the ratio of coal pitch to PSA resin, which is 1:1, the binary binder is prepared by the melt mixing method.

### 2.3. Preparation of Cf/C-SiC Composites

**Figure 1:**
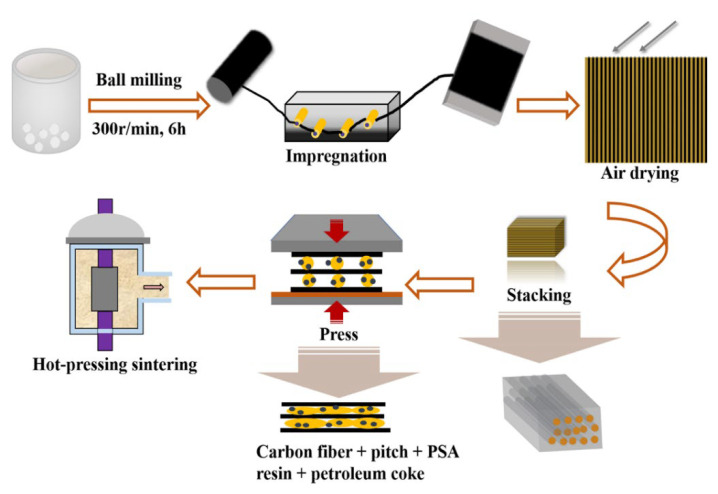
The schematic of the fabrication process of the Cf/C-SiC composites.

Cf/C-SiC composites were fabricated by slurry-impregnation and hot-pressing sintering processes in Figure 1. First, the slurry consisting of petroleum coke, binary binder, and semi-ceramic products of PSA was fabricated by ball milling process with deionized water as a solvent for eight hours. Polyethylene glycol, triton X-100, and methylcellulose were also added to adjust the viscosity and stability of the slurry, respectively. The detailed composition of each component in the slurry is shown in Table 1. In the current route, the use of semi-ceramic PSA powders as starting materials was considered not to influence the pyrolysis behavior of the binary binder because semi-ceramic PSA powder was an inert filler for the binary binder. Then, the infiltrated fibers were obtained using a filament winding machine by the slurry with a concentration of about 0.67 g/mL. After drying, the infiltrated fibers were cut into flakes with a size of 40 × 60 mm^2^ and then pressed into green compacts in the die at about 300 °C and 1 MPa pressure. Finally, it was conducted in a vacuum furnace and sintered at 1400 °C for one hour with a pressure of 10 MPa. Composite samples were designated as Cf/C-SiC-50%, Cf/C-SiC-60%, and Cf/C-SiC-70%, and 50%, 60%, and 70% represent the weight percentage of the binder reinforcement. The fiber volume fraction of the Cf/C-SiC composites was about 35%.

### 2.4. Mechanical Tests

The flexural strength of Cf/C-SiC composites (specimen size 3 mm × 4 mm × 40 mm) was measured using a three-point flexural approach in the Instron5967 (Instron, Boston, MA, USA) universal test machine. In this work, the span length was set as 30 mm, and the loading rate was 0.5 mm/min. The six testing specimens with a specified size were prepared by an inside diameter slicer. The flexural strength was calculated by the following equation, and the average value was taken:

(1) $$\sigma_{f}=\frac{3\mathrm{PL}}{{2\mathrm{bh}}^{2}}$$

where *σ*_f_ is the flexural strength (MPa); *P* is the maximum load (*N*); *L* is the span (mm); *b* and *h* are the width and depth of sample (mm).

The elastic modulus of composites can be calculated by the slope method according to the stress–strain curve in the bending strength test. The calculation formula of elastic modulus is:

(2) $$E=\frac{\Delta {\mathrm{PL}}^{3}}{{4\mathrm{bh}}^{3}\Delta s}$$

where *E* is elastic modulus (GPa); ∆*p*/∆*s* is the slope of the load–displacement curve; *L* is the span (mm); *b* and *h* are the width and depth of sample (mm).

### 2.5. Characterization

The FT-IR spectra of PSA resin and binary binder were obtained by the Fourier transform infrared spectrometer (Thermoelectric Nigaoli Instrument Co., Ltd., Madison, WI, USA), and the resolution was 4 cm^−1^ from 400 cm^−1^ to 4000 cm^−1^. The pyrolysis mechanism of samples was investigated by thermo-gravimetry (TG) and differential scanning calorimetry (DSC). The pyrolysis products of the samples were analyzed in a TG-MS analyzer (Thermo plus EV2, Bodong enterprise Co., Ltd, Shanghai, China), and the gas flow rate was 300 mL/min. X-ray diffractometer (XRD-6000, Cu Kα, 40.0 KV, 30.0 mA) ( Shimadzu enterprise management Co., Ltd., Tianjin, China) and Raman (HR Evolution Horiba, Tianjin Dongfang Kejie Technology Co., Ltd., Tianjin, China) were identified the phase structures of the samples. All samples were characterized as fired without surface polishing, and the microstructure of the Cf/C-SiC composites was observed by scanning electron microscopy (SEM, SU-8010, Hitachi Ltd., Shimane, Japan), and attached energy-dispersive spectroscopy (EDS) was used to analyze the elemental composition of the composites. The surface area and pore diameter measurements of Cf/C-SiC composites were measured by Brunner–Emmet–Teller (BET) equipment (Mac Instruments Co., Ltd., Atlanta, GA, USA), with nitrogen gas adsorption–desorption at −196 °C.

The apparent porosity and bulk density of Cf/C-SiC composites were measured according to Archimedes’ principle.

## 3. Results and Discussion

### 3.1. Characterizations of Binary Binder

**Figure 2:**
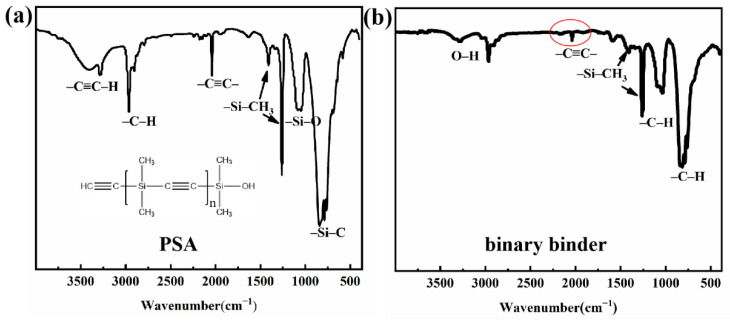
FT-IR spectra of (**a**) PSA and (**b**) binary binder (red circle is attributed to –C≡C–bond).

The synthesis process of PSA resin has been completed in the previous discussion [22]. PSA resin mainly includes –C≡C– bond and Si–CH_3_ bond, which form the {–[(CH_3_)_2_–C≡C]_n_} unit of the molecule. As shown in Figure 2, there are two typical peaks of PSA in the FT-IR spectra of binary binder, which can be attributed to the –Si–CH_3_– bond at 1258 cm^−1^ and –C≡C– bond at 2049 cm^−1^ [23]. Compared to PSA, the peak of –C≡C– appears weak in binary binder owing to the addition reaction for unsaturated alkenes between PSA and coal pitch. Moreover, the absorption in the range of 1350–1460 cm^−1^ is related to the bending vibration of the –C–H group [24]. The peaks at 700–900 cm^−1^ belong to the stretching vibration and out-of-plane bending vibration of –C–H in coal pitch aromatic structures [25,26], while the broad peak at 3291 cm^−1^ is attributed to the stretching vibration of O–H.

**Figure 3:**
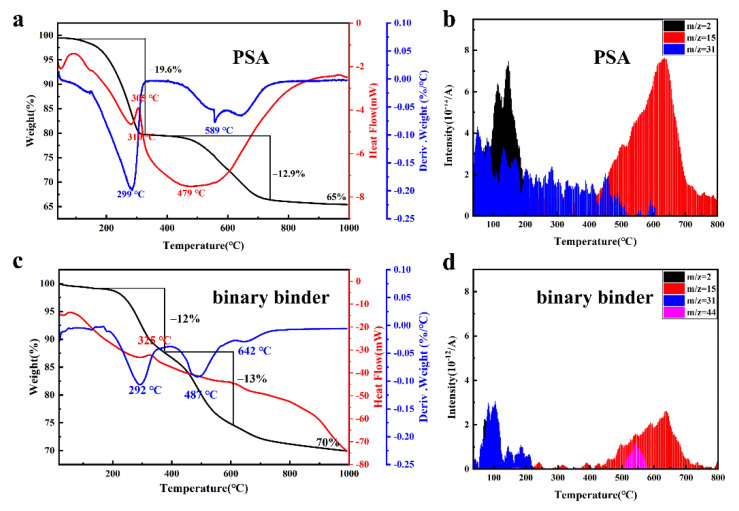
(**a**) TG-DSC-DTG curves of PSA resin; (**b**) MS spectrum of PSA resin; (**c**) TG-DSC-DTG curves of the binary binder; (**d**) MS curves of the binary binder.

To better understand the pyrolysis behavior, Figure 3 shows the TG-DSC-DTG and MS curves of PSA and the binary binder. PSA has three main mass-loss regions from 100 °C to 1000 °C in Figure 3a,b, and the largest mass-loss was located within 100 °C to 300 °C, with a value of 19.6%, owing to the escape of small molecules. Although there are also three main mass-loss regions of the binary binder in Figure 3c,d, the mass-loss is not sharp during pyrolyzation. In detail, (1) the region from 100 °C to 330 °C is attributed to the volatilization of water molecules and the loss of small molecules, such as solvents and oligomers, releasing part of H_2_ (*m*/*z* = 2) and CH_3_–CH_4_ (*m*/*z* = 31) [27]; (2) the region from 330 °C to 700 °C has the largest weight loss, including the decomposition of chain hydrocarbons, dehydrogenation, condensation, and polymerization of chain hydrocarbons, etc., taking place at the same time and release a large number of CH_3_^+^ (*m*/*z* = 15) and C_3_H_8_ (*m*/*z* = 44) gas [28]; (3) from 700 °C to 1000 °C, a very small weight loss is 2 wt% with a residue of 70 wt%. According to the result, the weight loss of the binary binder is relatively uniform during the whole pyrolysis process, and there is no short-term violent release stage (mass-loss stage), which causes excessive porosity of the composite.

### 3.2. Cf/C-SiC Composites Characterization

**Figure 4:**
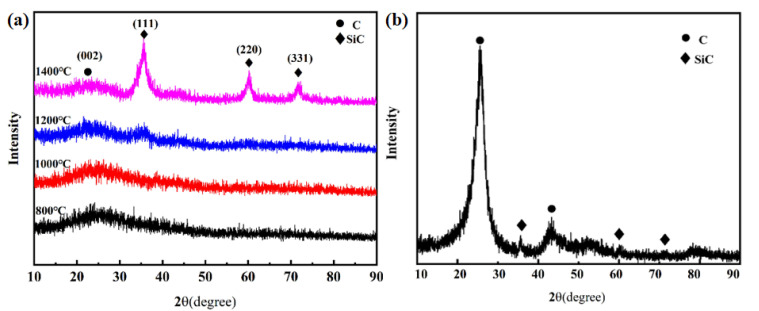
XRD pattern of samples: (**a**) the pyrolysis products of PSA at different heat-treatment temperatures; (**b**) the Cf/C-SiC composite after hot pressing at 1400 °C.

XRD pattern indicates that the amorphous PSA matrix converted to SiC ceramic at 1400 °C in Figure 4a. The peaks at 26.6°, 41.5°, and 43.4° are attributed to the lattice plane (002), (100), and (101) of SiC (JCPDS card no. 73-1708), respectively. Compared to the pyrolysis products of PSA, the Cf/C-SiC composites sintered at 1400 °C appear at two different crystalline phases, which indicates that the composite is mainly composed of C and SiC phases in Figure 4b. The characteristic peak at 26.4° is derived from the presence of carbon fibers and coal pitch and pyrolyzed Petroleum coke, while the other wide and low peaks belong to the SiC. Therefore, with the binary binder, the Cf/C-SiC composites could be manufactured successfully.

Table 2 shows the results of four composites with various content of binary binder. The overall sintering density of Cf/C-SiC composite is relatively low, which is due to the low crystallinity of SiC and certain closed pores during hot pressing. With the increase in binary binder content, the density of Cf/C-SiC composites gradually increases, and the open porosity firstly decreases and then increases.

**Figure 5:**
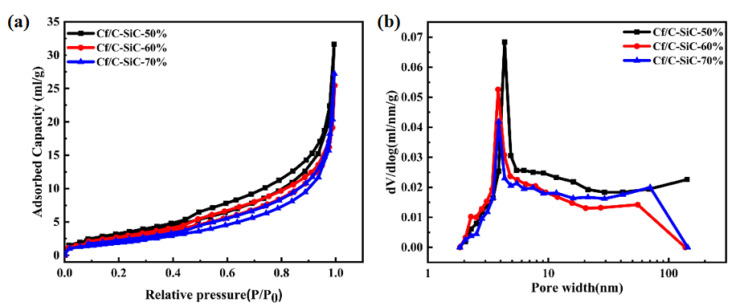
(**a**) Nitrogen sorption isotherms; (**b**) BJH pore size distributions of the Cf/C-SiC composites prepared by different binder content.

To further verify the reason for the low sintering density of composites, N_2_ isothermal adsorption–desorption was tested. The isothermal nitrogen adsorption and desorption curves and BJH pore size distribution curves of the composites with binder contents of 50%, 60%, and 70% are shown in Figure 5. All samples have the feature of IV-type curves with hysteresis loops, indicating a typical characteristic of mesopores. At high relative pressure (P/P0 > 0.8), nitrogen amounts absorbed rise steeply, indicating that some macropores are also present. The dV/d(logD) ordinate of the samples has a sharp peak around 3–5 nm, indicating that the composites contain a large number of mesoporous pores mainly concentrated in the vicinity of 3–5 nm. With the increase in binder content, the peak value of mesoporous pore size moves to the left, indicating that the increase in binder content leads to the decrease in pores between fiber bundles and the closer bonding between fiber and matrix. The detailed data of the specific surface area and pore size of all samples are shown in Table 3. From the pore structure parameters, it can be concluded that the average pore diameter of Cf/C-SiC-60% composite is the smallest, which is 12.95 nm.

Table 4 shows the effects of binary binder content on the mechanical properties of Cf/C-SiC composites. From these figures, the influence of binary binder content is apparent. The bending strengths of the Cf/C-SiC composite first increase and then decrease with the increase in binary binder content. In contrast, the Cf/C-SiC-60% composite has a relatively high bending strength, reaching a maximum of 339 ± 21 MPa at 1673 K and the maximum elastic modulus of 57.104 GPa.

As is known to all, the density and porosity of composites can influence the final mechanical properties of composites [29]. The opening rate decreases with the increase in binary binder content, which is due to the fulfilling of the pulp between the fiber bundles and the reduced pores. However, the Cf/C-SiC-70% composites show relatively low bending strength, which is due to the introduction of excessive binary binders, which leads to a large number of additional closed pores in the fiber. With the increase in matrix density, micro-cracks, holes, and other defects occur in the matrix. These defects lead to a longer stress transfer area and overstress range, and the load cannot be effectively transferred to the fiber, which inevitably reduces the uniformity of the microstructure of the composites and affects the mechanical properties of the composites [30].

**Figure 6:**
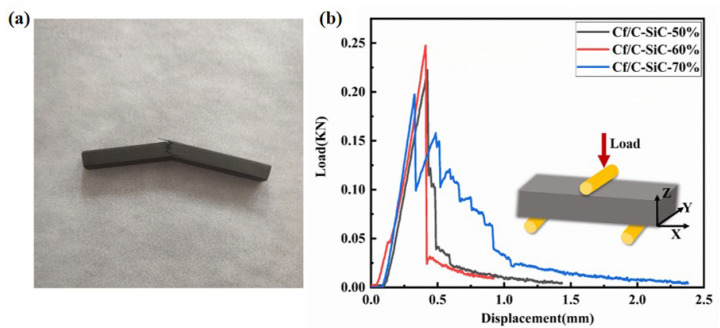
(**a**) Macromorphology of composites after three-point bending test; (**b**) the load–displacement curves of the Cf/C-SiC composites during the three-point bending test.

In Figure 6a, the macroscopic failure morphology of the bending specimens of Cf/C-SiC composites presents a bending shape, and the typical load–displacement curves for different specimens are depicted in Figure 6b. The stress–strain curve shows a nearly linear behavior when the applied stress reaches the maximum. After that, all of the curves gradually drop the load and extend long tails. The reason is that the matrix carries a more external load with the increase in load, which leads to crack propagation in the matrix. As the load further increased, more and more fibers gradually carried the load transferred from the matrix and finally made the fiber breakage. When the ratio of broken fibers reaches a critical value, the composites will fracture, which is given by the single fracture stress distribution. Because of the matrix cracking and fiber fracture, the nonlinear behavior can be seen in the load–displacement curve. Compared with Cf/C-SiC-50% composites, the load of Cf/C-SiC-60% composite increases sharply to the maximum with the increase in displacement and presents a steep linear slope at the initial stage, which may be due to the increase in the interfacial bonding strength between the fiber and the matrix caused by the increase in the binder content. For Cf/C-SiC-70% composites, the load–displacement curve reaches the peak and passes through a long sawtooth region. This is due to multiple mechanisms, such as crack deflection and crack branching.

The SEM images of a polished surface of Cf/C-SiC composites prepared by the HP process are shown in Figure S2. It can be seen that the carbon-fiber-reinforced materials are evenly dispersed in the matrix in the horizontal direction, and the fiber bundle was basically filled with the slurry. In comparison, Cf/C-SiC-50% composites have more pores, which is consistent with the previous porosity results. This is due to the low binder content during HP, which is not enough to fill the air gap in the CF region. Relatively speaking, the pore size in the CF region in Cf/C-SiC-60% and Cf/C-SiC-70% composites gradually decreases. In order to explore the distribution of the SiC phase in the composites, the Mapping analysis of Cf/C-SiC-60% composites was carried out in Figure S3, and the detailed information is shown in Table S1. The results show that the C element is the most, Si is the second, the O element content is the least, and the Si element is uniformly distributed in Cf/C-SiC composites. Therefore, the slurry impregnation-hot pressing sintering method can introduce the SiC phase into C/C composites, which solves the problem of uneven distribution of the SiC phase in the composites and improves the preparation efficiency of Cf/C-SiC composites.

Except for the different binder content, the raw materials and processing conditions used in all composites prepared in this experiment are mostly the same. It can be reasonably assumed that the difference in mechanical properties of composites caused by the change of binder content may be caused by the bonding strength of fiber/matrix. A large number of studies show that the bonding strength between fiber and matrix has a significant effect on the mechanical properties of the composites. It is generally believed that the proper interface bonding can significantly improve its flexural strength [31].

**Figure 7:**
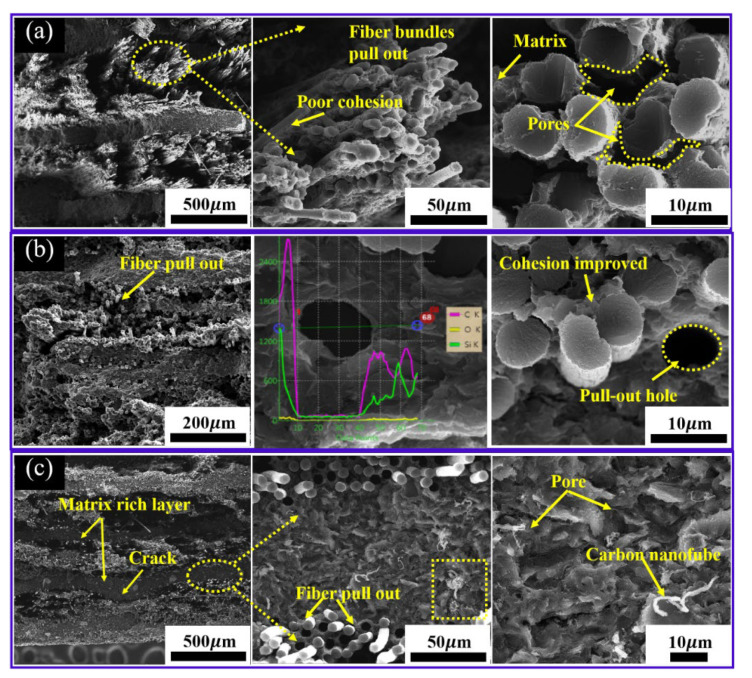
SEM images on the fracture surfaces: (**a**) Cf/C-SiC-50% composite; (**b**) Cf/C-SiC-60% composite; (**c**) Cf/C-SiC-70% composite.

Figure 7 shows the morphologies of the fracture surface of Cf/C-SiC composites with different binary binder content after the three-point flexural test. For Cf/C-SiC composites with continuous filament-winding molding and HP sintering, the laminate units are closely connected in the horizontal direction. In addition, due to the low sintering temperature, the chemical interaction at the fiber–matrix interface is inhibited so that the integrity of carbon fiber can be preserved successfully. As shown in Figure 7a, the cross-sections of Cf/C-SiC-50% composite are uneven, and two kinds of pores are observed. One is distributed in the carbon fiber bundles, and the other is distributed in the matrix area. This is because the low binder content cannot be enough to completely fill the interior of the fiber bundles, which makes it possible for the matrix to peel off the carbon fibers under the load. This is consistent with the bending load–displacement curve shown in Figure 6b. For the matrix part, the liquid binder cannot be enough to ensure the fluidity of semi ceramic PSA and petroleum coke. In addition, the binary binder releases a large amount of gas in the process of HP, which easily causes micro-cracks and a large number of holes. It is obvious that there are a large number of fibers pulled out in the form of fiber bundles on the fracture surface, and the length is longer, leaving holes, which means that the interfacial bonding strength between carbon fiber and matrix of Cf/C-SiC-50% composite is very weak, so the fiber could be easier to be pulled out under the load.

Obviously, for Cf/C-SiC-60% composite, no obvious fiber bundle pull-out is observed, while a small number of fibers are pulled out, and the fiber pull-out length is significantly lower than that of Cf/C-SiC-50% composites (Figure 7b). In addition, the carbon fiber bundles are tightly filled by the matrix phases without evident voids, which means that the combination of fiber and matrix is relatively tight. In order to deeply understand the fiber–matrix interface area, EDS line scanning is carried out along the central area of the cross-section of the left fiber hole. Figure 7b shows the Si distribution along the red line, indicating that there were no chemical reactions in the fiber–matrix interface. The O-content in fibers and matrix seems to remain at a low level from the Okα line scan. Benefitting from the uniform phase distribution and appropriate fiber/matrix bonding strength in the composites, the excellent flexural strength of Cf/C-SiC-60% composites is obtained.

If the binder content continues to increase to 70%, the bonding between the fiber and matrix will be closer (Figure 7c), indicating that a strong interfacial bonding has been developed in the composite. Due to the excessive content of the binder, it could generate volatile compounds and form voids in Cf/C-SiC during thermal treatment, which is also fully demonstrated by some carbon nanotubes in the matrix. The more defects in composites, the easier carbon nanotubes are grown. It can also be seen that the Cf/C-SiC-70% composites have more holes and micro-cracks in the matrix, which is consistent with the decrease in mechanical properties.

**Figure 8:**
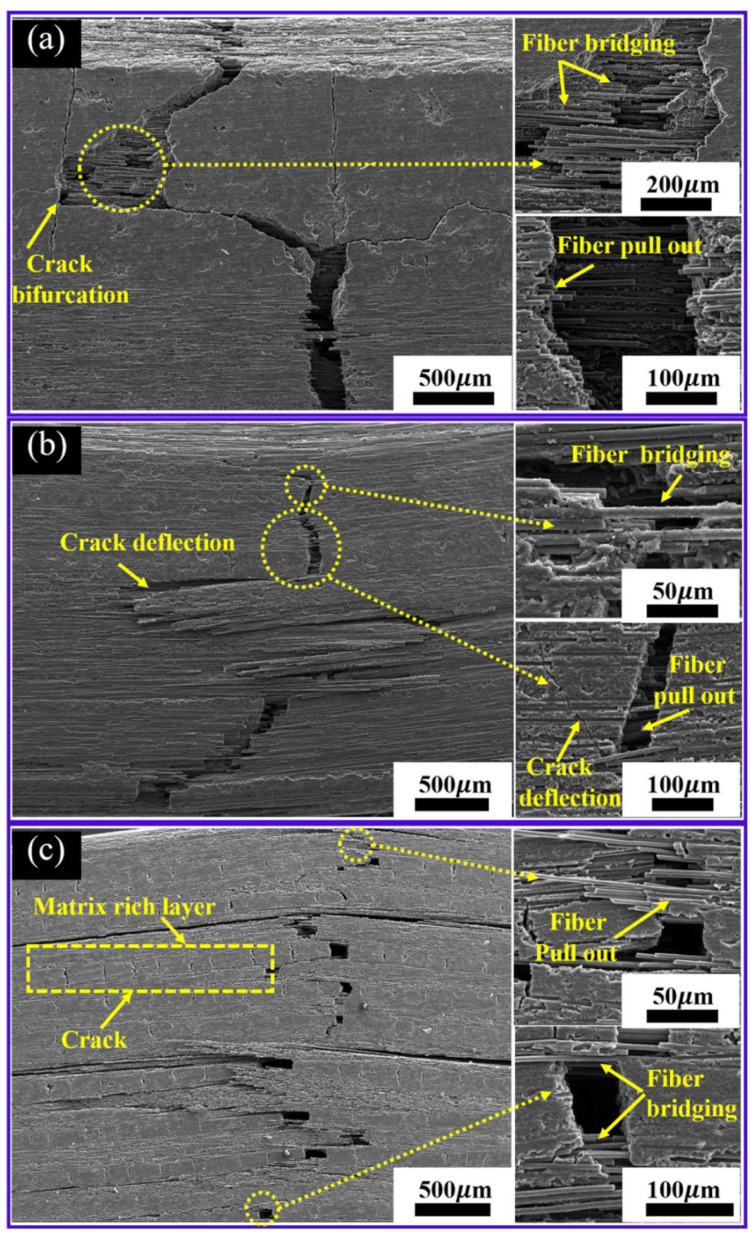
SEM images showing the crack patch of flexural tested composites: (**a**) Cf/C-SiC-50% composite; (**b**) Cf/C-SiC-60% composite; (**c**) Cf/C-SiC-70% composite.

The typical crack propagation path in the longitudinal sections of the composite after the three-point bending test was presented in Figure 8. The crack propagation path was tortuous as a whole. A large number of crack deflection and matrix breaking are observed, which is mainly related to the bonding strength of fiber/matrix and the thickness of the matrix layer. In the Cf/C-SiC-50% composite (Figure 8a), it is observed that the crack first starts with the carbon matrix. Due to the weak bonding strength between fiber and matrix, the crack can easily be expanded, and the deflection of the crack propagation was carried out along the fiber–matrix weak interfaces. Because of the low binder content, there are fewer matrices formed inside the fiber bundles, and the bonding between fibers is very weak, which will lead to the preferential propagation of cracks in the fiber bundles. The crack presented a zigzag path of propagation as a whole. A large amount of fiber pull-out (the longest pull-out length) and fiber bridging were also observed through the crack propagation path. As shown in Figure 8b, the fibers of Cf/C-SiC-60% composites have a more uniform matrix distribution, and the thickness of the matrix is significantly less than compared to Cf/C-SiC-50% and Cf/C-SiC-70% composites. If the thickness of the matrix is relatively large, the whole will behave like a ceramic and suddenly fracture and fail. In addition, the extension and branching of cracks and shorter fiber pull-out and fiber bridging were also observed.

In addition, when the binder content is too high, the binder completely fills the pores between fiber bundles. Under the action of pressure, the binder part is concentrated between the fiber stack and the stack to form a thicker matrix layer. When subjected to load, the thick matrix will present a brittle fracture mode similar to traditional ceramic materials, resulting in cracks perpendicular to the fiber direction. Crack deflection or branching occurs only if part of the thick matrix layer penetrates to the junction with the fibers. It is noted that the matrix thickness of Cf/C-SiC-70% composite is the largest, and more micro-cracks are in a rich matrix layer (shown in Figure 8c). These results show that matrix cracks are the main reasons for the increase in porosity. Additionally, these micro-cracks on the surface of the matrix may also cause the evident decrease in bending strength at room temperature. Almost all the directions of the cracks were perpendicular to the axial direction of the fiber in the matrix, so the transfer capacity of the matrix decreased. As the fiber and matrix are through Van der Waals forces and chemical bonds interaction, it was the weakest link. With the increase in load, this weakness would lead to the propagation of a large number of transverse cracks, resulting in interface delamination, fiber pull-out, and breaking. Micro-cracks in composites are usually considered the stress concentration point, and there are three main ways to account for micro-cracks generation: The carbonization process of overmuch coal pitch may also cause cracks, and the coal pitch itself contains impurity heteroatoms such as N and O, which will affect the generation of new carbon skeleton [32]. The conversion of the PSA in an amorphous SiC at a high temperature was usually accompanied by a large volume shrinkage. In the process of carbonization, the surface of petroleum coke becomes chemically inert, which is difficult to form a strong coupling with coal pitch carbon and SiC.

The above analysis indicates that the binary binder had a vital influence on the mechanical properties of the Cf/C-SiC composites. On the one hand, an appropriate amount of binary binder is helpful for the appropriate enhancement of the interfacial bonding degree between fiber and matrix, which is beneficial to improving the mechanical properties of composites. On the other hand, too much or too little binary binder will cause defects sun as the pores and cracks in varying degrees so that the flexural strength of the composite material is decisively reduced. Table 5 summarizes the published literature describing the Cf/C-SiC composites prepared by different preparation methods. It indicates that the Cf/C-SiC composites in this work show a lower density and higher bending strength. Considering the preparation process of the whole composite, increasing the yield of the binary binder and/ or the change of SiC content in the composite may be an effective way to further improve the mechanical properties of the composite. This work is currently underway.

## 4. Conclusions

In this work, a new binary binder was prepared by mixing PSA resin and coal pitch. PSA resin and coal pitch are partially cross-linked during the mixed heating pretreatment process. Due to the synergistic effect of the two, the low molecular weight volatile components were uniformly released during the whole pyrolysis carbonization process, and the residual yield was 70% at 1000 °C. Considering the compatibility between the various components of the composite, homogeneous precursor pyrolysis products were selected as fillers, which not only fill part of the pores but also effectively improve the content of SiC. The unidirectional continuous carbon-fiber-reinforced Cf/C-SiC composites were successfully prepared at low sintering temperature by a slurry impregnation-hot pressing process with a self-made binary binder. The 10 wt% SiC phase was effectively introduced, and a relatively uniform fiber/matrix distribution was constructed. We focused on determining the optimal amount of added composites binders for obtaining Cf/C-SiC composites with excellent mechanical properties. It is concluded that about 60 wt% of binary binders at 1400 °C was beneficial for the improvement of bending strength, and an excess of binary binders formed a large number of microcracks, leading to the decrease in bending strength. The mechanical properties of the composites sintered at 1400 °C under 10 MPa with 60 wt% binary binder content are the best, with a density of 1.53 g/cm^3^ and an average three-point flexure strength of 339 ± 21 MPa. SEM studied the microstructure and mechanical properties of Cf/C-SiC composites, and the bending strength mechanism and mode were analyzed. Overall, the excellent mechanical properties of these composites were attributed to the good fiber/matrix distribution and a desirable interfacial bonding. This work provided a facile and feasible way to fabricate Cf/C-SiC composites with good mechanical properties.

## Figures and Tables

**Table 1:** Proportion of components in slurry.

Samples	Component			Heat Treatment	SiC/%
	Binary Binder/%	Filler/%	Petroleum Coke/%		
Cf/C-SiC-50%	50	7.6	42.4	1400 °C 1 h	10
Cf/C-SiC-60%	60	3	37		
Cf/C-SiC-70%	70	0.5	29.5		

**Table 2:** Density and porosity of Cf/C-SiC composites with different binary binder content.

Samples	Binder Content (%)	Density (g cm^−1^)	Open Porosity (%)
Cf/C-SiC-50%	50	1.45	14.16
Cf/C-SiC-60%	60	1.53	1.28
Cf/C-SiC-70%	70	1.61	4.89

**Table 3:** BET test data of samples.

Sample	BET Surface (m^2^g^−1^)	Pore Volume (cm^3^g^−1^)	Pore Size (nm)
Cf/C-SiC-50%	11.8711	0.0428	14.4215
Cf/C-SiC-60%	9.9410	0.0322	12.9564
Cf/C-SiC-70%	7.4752	0.0346	18.5146

**Table 4:** Flexural strength of Cf/C-SiC composites with different binary binder content.

Samples	Bending Strength (MPa)	Elastic Modulus (GPa)
Cf/C-SiC-50%	256.5 ± 22	44.035
Cf/C-SiC-60%	339 ± 21	57.104
Cf/C-SiC-70%	217 ± 35	41.096

**Table 5:** Summary of the literature describing the Cf/C-SiC composites prepared by different preparation methods.

Samples	Method	Flexural Strength (MPa)	Ref.
Cf/C-SiC	HP (1437 K)	339 ± 21	This work
ERMI-C/C-SiC	ERMI (1673 K)	339	[14]
3-D Cf/C-SiC	CVI + RMI	170.72	[33]
Cf/SiC	PIP(PCS) + HP (1273 K)	138	[20]
2D-Cf/SiC	CVI+PIP(PCS) (SiC powder)	248	[17]
3D-braided C/SiC	PIP(PCS) + HP (1873 K)	490	[34]
Cf/C-SiC	LSI (1723 K)	195	[35]
3D needle-punched C/SiC	CVI + LMI	165	[36]
3D HTC C/C-SiC	CVI + PIP(PCS)	177.2	[37]

## Data Availability

Not applicable.

## Supplementary Materials

The following supporting information can be downloaded at: https://www.mdpi.com/article/10.3390/ma15082757/s1, Figure S1: SEM of pyrolysis products of PSA resin at 80 °C; Figure S2: (a) Schematic diagram of the molecular structure; (b) FTIR spectra of the polysilyacelene; Table S1: Detailed characterization of the raw pitch.
