# Supplementary material for: Binary Binder for Cf/C-SiC Composites with Enhanced Mechanical Property
Source: Materials (Basel). 2022 Apr 8;15(8):2757. doi: 10.3390/ma15082757 (PMC9030504; doi:10.3390/ma15082757)
Supplement: Supplementary file 1 [file materials-15-02757-s001.zip › materials-1661787-supplementary.pdf]

materials

Binary Binder for Cf/C-SiC Composites with Enhanced Mechanical Property

Yun Liu [1], Long Ma [1], Runa Dong [1], Kexin Cui [1], Yongzhao Hou [1,2,*], Wen Yang [3], Yeqing Liu [4], Cheng Zhong [1], Guangwu Wen [1,*] and Lijuan Zhang [1]

1 School of Materials Science and Engineering, Shandong University of Technology, Zibo 255000, China; ly17864301063@163.com (Y.L.); ml18340071781@163.com (L.M.); drn19121102039@163.com (R.D.); ckx9221@163.com (K.C.); aa1435884413@163.com (C.Z.); zhanglj@sdut.edu.cn (L.Z.)
2 Shandong Guiyuan Advanced Ceramics Co., Ltd., Zibo 255086, China
3 School of Transportation and Vehicle Engineering, Shandong University of Technology, Zibo 255000, China; yangwen004@sdut.edu.cn
4 Shandong Si-Nano Materials Technology Co., Ltd., Zibo 255400, China; liuyeqingyao@outlook.com
* Correspondence: houyz1990@sdut.edu.cn (Y.H.); wengw@sdut.edu.cn (G.W.); Tel.: +86-188-1630-7735 (Y.H.)

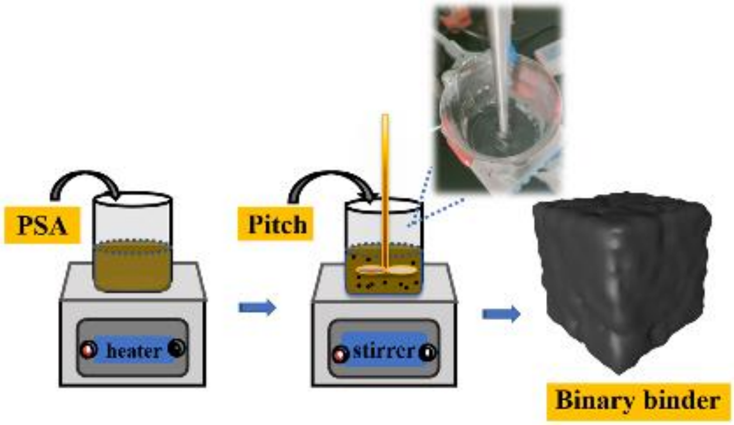

Figure S1. The schematic of the fabrication process of the composite binder.

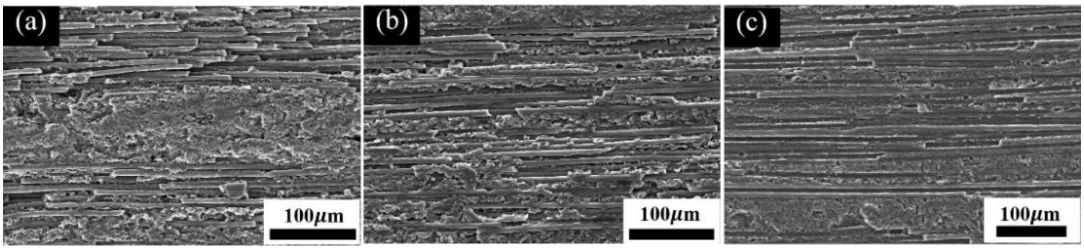

Figure S2. The microstructures of the polishing surface of the: (a) Cf/C-SiC-50% composite; (b) Cf/C-SiC-60% composite; (c) Cf/C-SiC-70% composite.

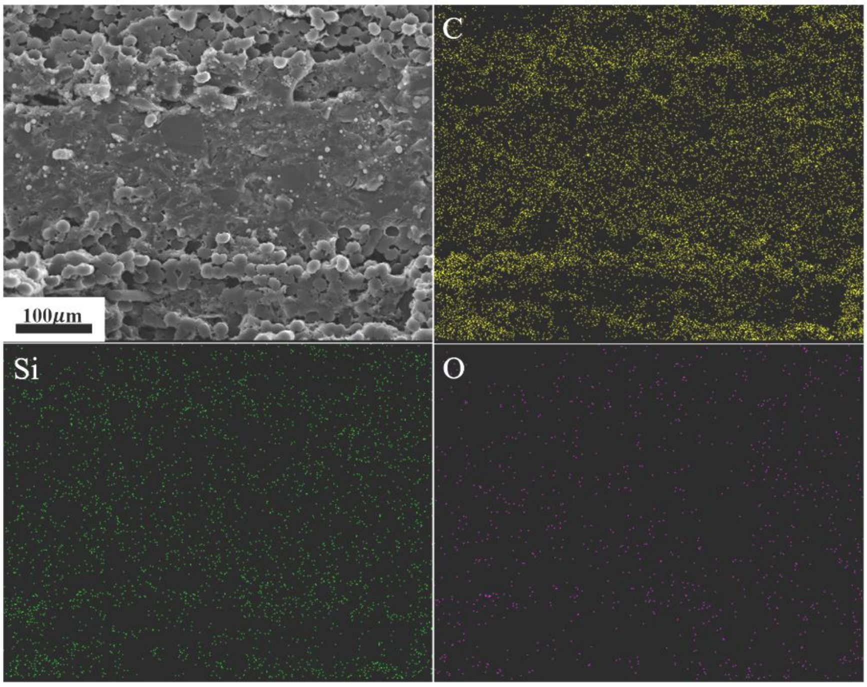

Figure S3. EDS analysis of Cf/C-SiC-60% composites.

Table S1. Map results of Cf/C-SiC-60% composite.

**Spectrum 1**			**Spectrum 2**		
**element**	**Wt(%)**	**At(%)**	**element**	**Wt(%)**	**At(%)**
Si	5.38	2.39	Si	5.39	2.39
C	92.13	95.67	C	92.11	95.66
O	2.5	1.95	O	2.50	1.95
